# Establishment of Functional PCR-Based Markers against Bacterial Leaf Blight Disease in Rice Landraces of Yunnan Province of China

**DOI:** 10.3390/life13102101

**Published:** 2023-10-23

**Authors:** Hengming Luo, Qun Wang, Chao Dong, Zhufeng Shi, Chengxing He, Zhixiang Guo, Junyi Shi, Chun Li, Wei Gao, Jinbin Li

**Affiliations:** 1 Agricultural Environment and Resource Research Institute, Yunnan Academy of Agricultural Sciences, Kunming 650205, China; lhm2670150914@163.com (H.L.); qunwang70@163.com (Q.W.); shizhfe@163.com (Z.S.); hechengxing69@163.com (C.H.); zhixiangg@163.com (Z.G.); shijunyi1003@163.com (J.S.); 2 Ministry of Agriculture and Rural Affairs International Joint Research Center for Agriculture, Kunming 650205, China; 3 Ministry of Agriculture and Rural Affairs Key Laboratory for Prevention and Control of Biological Invasions, Kunming 650205, China; 4 Yunnan Key Laboratory of Green Prevention and Control of Agricultural Transboundary Pests, Kunming 650205, China; 5 Biotechnology and Germplasm Resources Institute, Yunnan Academy of Agricultural Sciences, Kunming 650205, China; cchaodong@126.com; 6 Yunnan Seed Laboratory, Kunming 650205, China; 7 Yunnan Provincial Key Lab of Agricultural Biotechnology, Kunming 650205, China; 8 Key Laboratory of Southwestern Crop Gene Resources and Germplasm Innovation, Ministry of Agriculture and Rural Affairs, Kunming 650205, China; 9 Scientific Observation Station for Rice Germplasm Resources of Yunnan, Ministry of Agriculture and Rural Affairs, Kunming 650205, China; 10 Wenshan Plant Protection and Quarantine Station, Wenshan 663099, China; 13368767006@163.com (C.L.); gw_haim@163.com (W.G.)

**Keywords:** bacterial blight, resistance, functional markers, rice

## Abstract

Bacterial leaf blight is a devastating disease of rice worldwide. The resistant genes are routinely transferred from landraces to cultivated varieties through backcross breeding along with marker-assisted selection. In the present study, we use the gene-specific markers to screen the rice landraces in Yunnan Province of China. We collected 404 representative samples of 24 different rice landraces from Yunnan Province of China. The initial PCR-based screening suggested that the leaf blight resistance was not evenly distributed in Yunnan Province. Our results indicate that there is a complete loss of resistance for landraces based on *xa5* and *xa13* genes. On the other hand, landraces harboring *Xa7* and *Xa21* showed a high level of resistance. Using gene-specific PCR-based data, we were able to identify the resistant, susceptible and heterozygous populations across Yunnan Province. The widely used *Xa21* gene alone showed a remarkable level of resistance throughout the province, indicating its potential to develop broad-spectrum resistance in rice germplasm. The key aspects of bacterial blight spread according to local sites in Yunnan Province and the resistance conferred by different landraces due to the presence of different resistance genes are discussed.

## 1. Introduction

The bacterial pathogen Xanthomonas campestris pv. Oryzae (commonly referred to as “*Xoo*”) is the major cause of blight disease in rice [1]. The bacterial blight (BB) disease can cause more than 0% loss of grain yield in rice. The disease occurs primarily on leaves and can subsequently infect the stem, panicle and rice grains. It has been observed that high humidity due to continuous rainfall and warm temperatures can cause the rapid spread of the disease. The bacterium can survive for a long time in seeds, plant debris, or in the soil and is spread through water, rain splashes and infected seeds [2]. It is well-known that sever bacterial blight infection in rice can lead to complete crop failure and a reduction in grain size and their market value. However, in the recent past, the integrated disease control strategies involving marker-assisted selection, disease resistance gene introgression and genome editing technologies have been extensively used [3,4,5,6]. The invasion of *Xoo* occurs through the opening of leaf cells, which then spread to the xylem cells. The bacterium also requires some host genes, such as the thiG gene, for its virulence [7,8]. 

Three different approaches including biological control, systemic bactericides and genetic resistance have been effectively used to control the bacterial blight disease in rice [9,10]. Despite the use of integrated approaches, the use genetic resistance is considered to be the most effective and environmentally friendly way to control the bacterial blight disease in rice. Rice breeders have played a crucial role in the development of genetically resistant rice varieties. In fact, molecular breeding tools, mainly the marker-assisted selection have been instrumental in integrating resistant genes into commercial varieties [11]. In the extensive breeding efforts, several blast resistance genes like *Xa4*, *xa5*, *Xa7*, *xa13* and *Xa21* have been introduced into japonica rice cultivars [12,13]. Recent molecular breeding approaches have made it possible to develop advanced lines for economically important traits with accuracy and efficiency. The new breeding tools such as marker-assisted selection (MAS), genomic selection (GS), and marker-assisted backcrossing (MABC) have proven fruitful in detecting the origin and transfer of important traits in different cultivars. These gene pyramiding efforts using genomics tools and recurrent backcrossing resulted in bacterial leaf blight resistance in rice. 

To evade the host immune system, the *Xoo* weakens the host receptors or inhibits the cell signaling pathway. The resistant plants have evolved the immunity by diversifying the receptors and their recognition patterns [14]. The broad-spectrum receptors-based level of resistance in plants is also referred to as basal immunity. However, the gene-for-gene resistance is another level of broad-spectrum resistance. Both the mechanisms involve receptor kinases and nucleotides-binding leucine-rich repeats (NB-LRR) [15]. The *Xoo* rice interaction is a classic example of gene-for-gene resistance in the plant kingdom. The majority of resistance genes in rice are genetically recessive in nature. So far, forty-seven bacterial blight resistance (R) genes have been identified. These genes are well characterized by DNA-markers. However, several resistance genes like *Xa21*, *Xa7* and *xa5* have been cloned and characterized at molecular level [4,16,17]. Most of the cloned *R* genes encode nucleotides-binding and leucine-rich repeat domain (NLR) proteins. In fact, the first resistance gene (namely *Xa21*) conferring resistance against *Xanthomonas oryzae* was identified as a receptor from *Oryzae longistaminata* [18]. After the first identification of the *Xa21 R* gene from rice in 1995, it was transferred and introgressed into several cultivars by molecular breeding methods [19,20]. 

The literature suggests that *Xa21* or other resistance genes have revolutionized rice breeding worldwide. It is interesting to note that out of forty-seven known R genes, sixteen are known to be recessive genes, while few exhibit co-dominance as seen in the case of *Xa27* [21,22]. These fourteen genes have been well characterized for their genetic makeup and functions (Reviewed by Yang et al., [23]). These experiments revealed multiple mechanisms involved in conferring resistance against *Xoo* strains. Some of the R genes like, *Xa22*, *Xa23* and *Xa39* confer broad-spectrum resistance against a wide range of *Xoo* isolates, making them suitable for integration into molecular breeding programs. However, the experiments on resistant rice varieties suggest that the rapid evolution of *Xoo* can easily overcome the genetic resistance. Therefore, continuous efforts of resistance gene mining are a good approach to counter the evolution of pathogens. The genes like *Xa7*, *Xa26*, *Xa27* and *Xa21* were cloned by PCR-based amplification techniques and used in gene pyramiding breeding to develop broad-spectrum resistance in rice [24,25]. 

In the late 1970s, the rice production in China suffered from a major bacterial blight outbreak. However, with the introduction of blight-resistant varieties, the problem was quickly overcome. The introgression of the *Xa21* gene in the elite hybrid rice varieties, namely Minghui-63, resulted in the broad-spectrum resistance against six different races of *Xoo* [26]. While in India, the introduction of *Xa21* in the susceptible variety PR106 showed resistance to seventeen different races of *Xoo* [27]. However, trials in Vietnam suggested that some of the *Xoo* races can overcome the *Xa21* based resistance [28]. The R genes confer resistance to the rice blast disease, which is a major threat to rice crops. These genes are mostly found in different rice cultivars, landraces, and non-cultivated or wild rice lines. The major concern of the scientific community is the introduction and development of improved rice varieties, which have led to the replacement of conventional landraces and varieties, ultimately reducing the genetic diversity of cultivated rice. However, maintaining the genetic diversity is essential for adaptation to ever-changing environmental conditions and new pests. Although wild relatives and landraces may not possess desirable, important traits for modern breeding programs, they remain a valuable source of genes related to tolerance against biotic and abiotic stresses. In order to conserve the resistant germplasm and control diseases like rice blast, it is imperative to explore the blast resistance genes and develop the genetic markers. The development of molecular markers is an important and critical step in the breeding program, aimed at developing resistant rice varieties. The purpose of this study is to explore the current status of bacterial blight resistance in the local landraces of Yunnan Province through PCR-based DNA markers. 

In this study, by using PCR approaches, we were able to identify four blight resistance genes (*xa5*, *Xa7*, *xa13* and *Xa21*) in 404 rice landrace samples representing twenty-four different genotypes from Yunnan Province, China. Different levels of resistance and susceptibility were confirmed through our PCR-based markers. This is also the first large scale report on the distribution of blight disease in Yunnan Province, China.

## 2. Materials and Methods

### 2.1. Selection of Rice Landraces

In this study, we analyzed 404 samples collected from Yunnan Province of China. The landraces were collected from all parts of Yunnan Province. The leaf samples were labeled and stored in ice. The samples were kept frozen until further use in the laboratory. The distribution of the 24 landrace genotypes is shown in Table S1. The reference lines, IRBB5, IRBB7, IRBB13, IRBB21 and IR24 (Susceptible to all BB strains), were also used as susceptible lines in this study [29]. Unequal numbers of symptomatic plants for each landrace were collected from different parts of Yunnan Province during 2018. The differences in sample size were due to the availability of recognizable symptoms. Accession IET-8585 was used as a resistant line to validate the results [30].

### 2.2. DNA Isolation for Molecular Analysis of Resistant Genes

Total genomic DNA from resistant and susceptible landraces was isolated from field-grown rice landraces using a modified Cetyl Trimethyl Ammonium Bromide (CTAB) method as described previously [31,32]. Briefly, 0.5 g leaf samples were collected and ground to fine powder in the liquid nitrogen. Approximately, 700 µL of preheated CTAB was added and incubated at 65 °C for 30 min [33]. Chloroform isoamyl alcohol (24:1) was then added and incubated at room temperature for 10 min. The samples were centrifuged at 12,000 rpm and the supernatant was collected in a new Eppendorf tube. The 3M sodium acetate and 600 µL of ice-cold isopropanol were added. The samples were centrifuged at 12,000 rpm for 15 min and the DNA pellet was air-dried. Finally, nuclease free water was added to dissolve the pellet and ~25 ng/µL final concentration was used in PCR reactions to analyze *Xa21*, *xa13*, *Xa7*, and *xa5* resistance genes.

### 2.3. Amplification of Resistance Genes

For amplification of resistant genes (*xa5*, *Xa7*, *xa13* and *Xa21*), the specific universal primer sets were used in 25 µL PCR reaction (Table 1; Vazyme, China). For each PCR reaction (volume 25 µL) 1 µL of each primer (10 µM) was used. The PCR program was as follows: initial one cycle at 95 °C for 5 min, followed by 30 cycles at 95 °C for 30 s, 55 °C for 30 s, 72 °C extension temperature for 60 s. A final extension at 72 °C for 10 min was used.

### 2.4. Marker-Based Screening for Bacterial Leaf Blight Resistance in Rice Landraces

Based on the PCR amplification of four resistance genes, (*xa5*, *Xa7*, *xa13* and *Xa21*), the percentage for resistant (R), susceptible (S), heterozygous or co-dominant (H) and absence of any R gene (-) was calculated. Each amplified product from landrace accession in the PCR reaction was run on an ethidium bromide stained 1% agarose gel along with a 100 bp DNA ladder. Additionally, the distribution of R, S and H was also calculated for all the collected landraces from each part of Yunnan Province, including eastern, western, northern, southern, central, northwestern, and southeastern regions.

## 3. Results

### 3.1. Resistance and Susceptibility Levels of Rice Landraces to Bacterial Leaf Blight

All four of the primer pairs showed consistent amplification among susceptible and resistant landraces. Therefore, these polymorphic primer pairs were used to validate the resistance or susceptibility of different rice landraces. The functional marker pTA248 for *Xa21* was able to discriminate resistant and susceptible landraces. Similarly, the functional markers for *xa13* (xa13-prom), *Xa7* (Xa7STSP3) and *xa5* (xa5FM) were developed to clearly distinguish the resistant, susceptible and heterozygous plants of different landraces. The marker-based screening results revealed that majority of landraces were susceptible (Table 2). The highest percentage of resistant alleles (R) were observed in the genotypes harboring the *Xa21* gene (38.6%). In contrast, the genotypes with the *xa5* and *xa13* genes did not show the presence of R alleles. In fact, out of 404 landraces analyzed here for *xa13*, 398 samples showed the presence of susceptible alleles. These samples were also homozygous in nature. On the other hand, the functional marker xa5FM showed the presence of either susceptible or heterozygous alleles. The representatives of landraces harboring *xa5* had the highest number of heterozygous plants (37.9%), whereas all the *xa13* plants were homozygous in nature (Table 2). It is obvious that landraces with the *Xa7* and *Xa21* genes showed a broad-spectrum resistance against bacterial blast disease of rice. 

### 3.2. Molecular Detection of R Genes in Landraces

**Figure 1:**
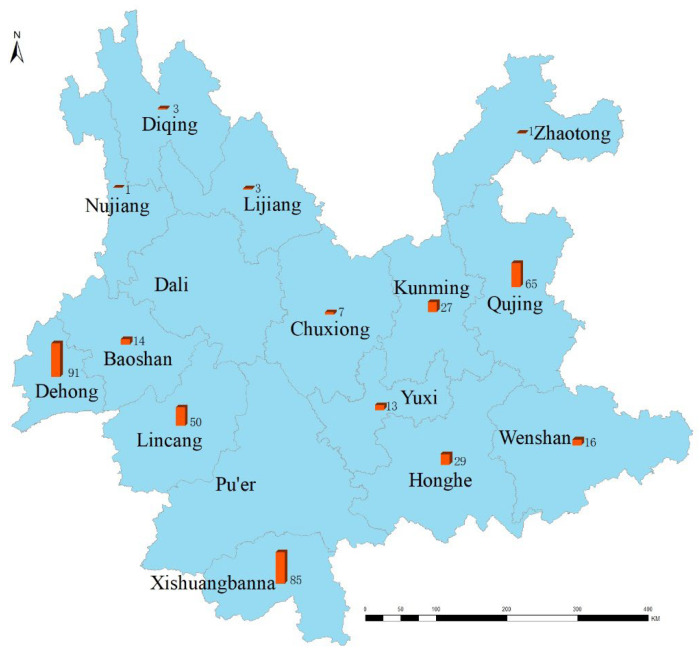
The site locations selected for sample collection. The exact location and number of samples are mentioned for areas of Yunnan Province in China.

**Figure 2:**
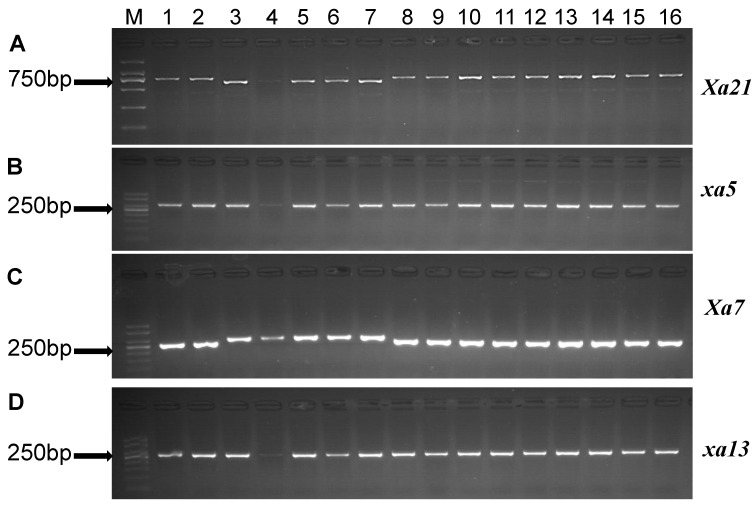
Amplification patterns of polymorphic markers pTA248, xa5FM, STSP3 and xa13-prom. PCR products amplified from genomic DNA of representative rice landraces, (**A**) marker pTA248 for *Xa21*, M = DL 2000 DNA ladder, (**B**) marker xa5FM for *xa5*, M = 50-bp DNA ladder, (**C**) marker STSP3 for *Xa7*, M = 50-bp DNA ladder, (**D**) marker xa13-prom for *xa13*, M = 50-bp DNA ladder. Lanes 1–16 represent representative samples of rice landraces collected from Yunnan Province.

We used marker-based PCR for the confirmation of R genes at molecular level. In fact, the molecular marker-based results for resistance genes were consistent with the phenotypic data. The samples analyzed from different parts of Yunnan Province showed differences in the distribution of resistance (Table 3, Figure 1 and Figure 2).

**Figure 3:**
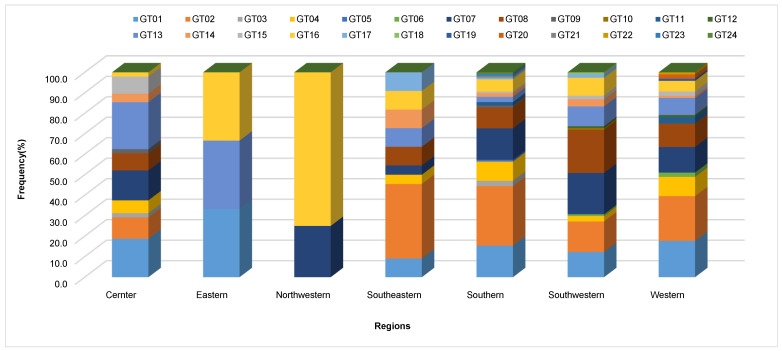
The distribution of genotypes with resistance genes in landrace collected from different regions of Yunnan Province, China.

The PCR amplification of the *Xa21*, *xa5*, *Xa7* and *xa13* genes revealed that the majority of rice samples have functional pTA248, xa13prom and STSP3 markers [34,37]. The results indicated that ~750 bp band for *Xa21* marker was amplified, while, for *xa5* and *Xa7*, approximately 280 bp and 250 bp bands were amplified (Figure 2). Similarly, a band of ~280 linked to *xa13* marker was amplified. Molecular analysis indicated that the *Xa7* and *Xa21* R genes were widely distributed in central (37.5 and 11%) and southwestern (26.3 and 41.2%) regions, while the southeastern region was mainly dominated by *Xa21* R gene (63.4%). However, in the eastern region, none of the plants showed any level of *Xa21* R gene amplification (Table 3 and Figure 3). It is noteworthy that the germplasm carrying *Xa21* and *Xa7* was found to be resistant. The plants showing susceptibility despite of the presence of *Xa21* and *Xa7* (Table 3) can be related to the higher pathogenicity level of *Xoo* strains [38,39,40]. Remarkably, even the presence of single allele *Xa7* or *Xa21* showed a complete level of resistance (Table 3). However, the germplasm carrying *xa5* and *xa13* were highly susceptible and did not show any detectable level of resistance.

### 3.3. Characteristics of the Distribution of 4 Bacterial Blight Resistance Genes in Landraces

We tested whether the twenty-four genotypes we selected show any variation in the blight resistance (Table 4). The data suggested that the first twelve genotypes (GT1–GT12) were susceptible in the presence of *Xa7* locus, whereas none of the genotypes with *xa13* locus showed resistance to blight disease. As expected, the genotypes harboring *xa5* and Xa21 genes showed variation in the resistance levels; the majority of the genotypes were resistant to blight disease. The genotypes GT01, 4, 5, 11 and 12 did not show any level of resistance; therefore, these should not be included in the breeding program. The presence of heterozygous (H) and resistance (R) genes suggests that the genotypes 8, 14, 17 and 20 can be further used in gene pyramiding experiments.

## 4. Discussion

Rice is the main staple food for almost half of the world’s population. Its grain yield is highly dependent on biotic and abiotic factors. Among these factors, the pathogenic organisms such as bacteria and fungi pose a serious challenge to rice production [41]. It is an established fact that genetic approaches to incorporate broad-spectrum resistance against rice pathogens can prevent the yield losses [42]. In fact, a significant number of resistance genes associated with bacterial blight disease have been identified; however, due to the emergence of highly pathogenic strains, the resistance developed through various breeding approaches is at higher risk [11,43]. The incorporation of R genes into rice is an effective and sustainable strategy to manage the bacterial blight in rice. Together with marker-assisted selection and modern molecular tools, R gene stacking into elite but susceptible germplasm has achieved the goal of disease resistance in rice. With these efforts, forty-seven different R genes have been characterized to confer resistance against *Xoo* strains or races [44]. Although a large number of R genes are known, among them *Xa4*, *xa5*, *Xa7*, *xa13* and *Xa21* are most widely used to develop disease resistance in rice varieties. Among them, gene *xa5* is a well-known subunit of the transcription factor (TFIIA) [17]. The previous experiments suggested that *xa5* in combination with *Xa21* significantly improved the bacterial blight resistance in the recipient lines [45]. In fact, the *Xa21* codes for NBS-LRR proteins, which are well known effectors molecules, target the virulence of pathogens. The pyramiding experiments have proven that the incorporation of such genes develops broad-spectrum resistance in rice [13]. These molecular markers can mainly facilitate the evaluation of disease resistance [46]. Previously, various studies reported the presence of resistance genes in rice landraces using molecular markers [35]. The literature suggests that in Yunnan Province, the phenotypic resistance to at least ten *Xoo* strains has been observed in the different landraces. Together with the previous observations and our analysis, it indicates that the presence of multiple R genes can develop a buffering resistance against several isolates of *Xoo* strains. Therefore, it further strengthens the concept that different landraces with multiple but unique R genes can be a valuable resource for developing disease resistance in rice. 

Due to the strong association of bacterial resistance in rice, we selected *xa5*, *Xa7*, *xa13* and *Xa21* genes in this study. China is one of the leading countries in the world in terms of rice production and Yunnan Province is considered as the center of diversity for rice genetic diversity and home to large number of indigenous rice landraces [47]. The rice landraces originating from China have shaped the evolution of bacterial blight resistance in cultivated varieties through the transfer of single resistance genes or in combination. There is consensus that a homozygous combination of resistance genes (*Xa21* and *Xa7*) develops durable resistance against *Xoo* strains [40]. In the gene pyramiding experiments, the transfer of a single gene in heterozygous condition developed incomplete resistance against *Xoo* strains. However, in homozygous conditions or hybrids comprising two different R genes, the strong resistance against bacterial strains was observed. Therefore, in our experiment it was observed that despite the harboring of a single R gene (*Xa21* or *Xa7*), the disease development was observed on landraces (Table 3). The hybridization and backcross breeding between the wild relative and domesticated crops ensured crop varieties had high phenotypic and genetic potential. In this study, 404 different samples comprising twenty-four different landraces of Yunnan were evaluated for bacterial blight susceptibility or resistance-related genes, namely, *xa5*, *Xa7*, *xa13* and *Xa21*. The samples were collected from seven different localities of Yunnan Province. Based on the data analysis, the genotypes harboring *xa5*, *xa13* did not show any level of disease resistance in any part of Yunnan Province. However, a significant number of plants harboring *Xa7* and *Xa21* showed a relatively high level of resistance. Our results are consistent with the previous studies that observe that *Xa21* confers resistance to several *Xoo* strains and can be effectively used for introgression into cultivated rice varieties [20,28,37]. The wide distribution of landraces harboring the *Xa7* and *Xa21* genes in Yunnan Province is plausible because they can better resist multiple *Xoo* pathotypes. Despite the fact that PCR-based authentication for *Xa21*, *xa5*, *xa13* and *Xa7* is reliable, the exact degree of heterogeneity and their frequency in the natural population is not known yet. Our results clearly indicate that although R genes *xa5* and *xa13* are present in the landraces, their presence did not show any detectable level of bacterial resistance [48,49].

Previously, *Xa21* has been used in breeding programs in different countries [26,50]. Among all resistant genes, the genotypes integrated with *Xa21* showed reliable broad-spectrum resistance. However, in our and other’s findings, despite the presence of *Xa21*, the bacteria overcome the natural resistance [50]. Due to the presence of several resistant genes, it is important to develop cost-effective functional markers for authentication in the breeding materials. Our PCR-based strategy effectively described the utilization of four resistant genes and their markers unambiguously. PCR-based markers for *Xa21*, *xa13* and *xa5* have been developed and functionally validated [34]. However, in the previous approaches, an additional step of restriction digestion was also used to develop functional polymorphic markers. The use of one-step PCR or single tube multiplex PCR is advantageous in terms of its ease of handling and sophistication in validity. 

## 5. Conclusions

Our study concluded that multiple resistant genes are present in rice landraces in Yunnan Province. However, the frequency of resistant and susceptible genes and their effects are variable in the region. However, the landraces with the R genes, namely *xa5* and *xa13*, are not resistant to the prevailing bacterial strains in the Yunnan Province. Multiplex marker-based screening can be a useful strategy to control the spread of disease.

## Figures and Tables

**Table 1:** Functional primer sequences for the *Xa21*, *xa13*, *Xa7* and *xa5* genes.

Gene	Primer Name	Primer Sequence	Reference
***Xa21***	PTA248 F	5′-AGACGCGGAAGGGTGGTTCCCGGA-3′	[34]
	PTA248 R	5′-AGACGCGGTAATCGAAAGATGAAA-3′	
***xa13***	xa13-prom F	5′-GGCCATGGCTCAGTGTTTAT-3′	[35]
	xa13-prom R	5′-GAGCTCCAGCTCTCCAAATG-3′	
***xa5***	xa5FM-SF	5′-GTCTGGAATTTGCTCGCGTTCG-3′	[35]
	xa5FM-SR	5′-TGGTAAAGTAGATACCTTATCAAACTGGA-3′	
	xa5FM-RF	5′-AGCTCGCCATTCAAGTTCTTGAG-3′	
	xa5FM-RR	5′-TGACTTGGTTCTCCAAGGCTT-3′	
***Xa7***	STSP3F	5′-CAGCAATTCACTGGAGTAGTGGTT-3′	[36]
	STSP3R	5′-CATCACGGTCACCGCCATATCGGA-3′	

**Table 2:** Genotypes based on the type and number of 4 bacterial blight resistance genes *Xa7*, *xa5*, *xa13*, and *Xa21* of rice detected by PCR.

	*Xa7*	%	*xa5*	%	*xa13*	%	*Xa21*	%
**No. of R**	91	22.5	0	0.0	0	0.0	156	38.6
**No. of S**	306	75.7	241	59.7	398	98.5	191	47.3
**No. of H**	7	1.7	153	37.9	0	0.0	7	1.7
**No. of** -	0	0.0	10	2.5	6	1.5	50	12.4
**Total**	404	100.0	404	100.0	404	100.0	404	100.0

Note: R: the resistant allele genotypes, S: susceptible allele genotypes, H: heterozygous allele individuals, -: absence of R gene.

**Table 3:** Characterization of landraces on the basis of PCR results of the *Xa7*, *xa5*, *xa13* and *Xa21* genes and diversity index of genotypes among landrace in Yunnan Province.

Regions	No. of Rice Accessions	Allele	Resistance Gene and Frequency							
			*Xa7*	%	*xa5*	%	*xa13*	%	*Xa21*	%
Center	48	No. of R	18	37.5	0	0.0	0	0.0	11	22.9
		No. of S	30	62.5	35	72.9	48	100.0	28	58.3
		No. of H	0	0	13	27.1	0	0.0	2	4.2
		No. of -	0	0	0	0.0	0	0.0	7	14.6
Eastern	3	No. of R	2	66.7	0	0.0	0	0.0	0	0.0
		No. of S	1	33.3	2	66.7	3	100.0	3	100.0
		No. of H	0	0.0	1	33.3	0	0.0	0	0.0
		No. of -	0	0.0	0	0.0	0	0.0	0	0.0
Northwestern	4	No. of R	3	75.0	0	0.0	0	0.0	0	0.0
		No. of S	1	25.0	0	0.0	4	100.0	4	100.0
		No. of H	0	0.0	4	100.0	0	0.0	0	0.0
		No. of -	0	0.0	0	0.0	0	0.0	0	0.0
Southeastern	22	No. of R	8	36.4	0	0.0	0	0.0	14	63.6
		No. of S	14	63.6	15	68.2	22	100.0	7	31.8
		No. of H	0	0.0	7	31.8	0	0.0	0	0.0
		No. of -	0	0.0	0	0.0	0	0.0	1	4.5
Southern	117	No. of R	14	12.0	0	0.0	0	0.0	49	41.9
		No. of S	100	85.5	74	63.2	116	99.1	48	41.0
		No. of H	3	2.6	39	33.3	0	0.0	4	3.4
		No. of -	0	0.0	4	3.4	1	0.9	16	13.7
Southwestern	114	No. of R	30	26.3	0	0.0	0	0.0	47	41.2
		No. of S	84	73.7	52	45.6	112	98.2	58	50.9
		No. of H	0	0.0	60	52.6	0	0.0	0	0.0
		No. of -	0	0.0	2	1.8	2	1.8	9	7.9
Western	96	No. of R	16	16.7	0	0.0	0	0.0	35	36.5
		No. of S	76	79.2	63	65.6	93	96.9	43	44.8
		No. of H	4	4.2	29	30.2	0	0.0	1	1.0
		No. of -	0	0.0	4	4.2	3	3.1	17	17.7
Total	404									

Note: R: the resistant genotypes, S: susceptible genotypes, H: heterozygous genotypes, amplified both the fragments in a co-dominant fashion, -: no PCR productions.

**Table 4:** Distribution of genotypes of 4 bacterial blight resistance genes in landraces collected from Yunnan, China.

Genotypes	Resistance Gene Allele				No. of Accessions	Frequency (%)
	*Xa7*	*xa5*	*xa13*	*Xa21*		
GT01	S	S	S	S	61	15.1
GT02	S	S	S	R	85	21.0
GT03	S	S	S	H	4	1.0
GT04	S	S	S	-	27	6.7
GT05	S	S	-	S	1	0.2
GT06	S	S	-	-	3	0.7
GT07	S	H	S	S	62	15.3
GT08	S	H	S	R	53	13.1
GT09	S	H	S	H	2	0.5
GT10	S	H	S	-	1	0.2
GT11	S	-	S	-	5	1.2
GT12	S	-	-	-	2	0.5
GT13	R	S	S	S	36	8.9
GT14	R	S	S	R	11	2.7
GT15	R	S	S	-	9	2.2
GT16	R	H	S	S	29	7.2
GT17	R	H	S	R	5	1.2
GT18	R	-	S	-	1	0.2
GT19	H	S	S	S	1	0.2
GT20	H	S	S	R	2	0.5
GT21	H	S	S	-	1	0.2
GT22	H	H	S	H	1	0.2
GT23	H	-	S	S	1	0.2
GT24	H	-	S	-	1	0.2
Total					404	100.0

Note: R: the resistant gene allele, S: susceptible gene allele, H: heterozygous individuals, amplify both the two fragments in a co-dominant fashion; -: no PCR productions.

## Data Availability

Not applicable.

## Supplementary Materials

The following supporting information can be downloaded at: https://www.mdpi.com/article/10.3390/life13102101/s1, Table S1: Distribution of genotypes of 4 bacterial blight resistance genes in rice landraces collected from different regions of Yunnan, China.
