# Supplementary material for: Establishment of Functional PCR-Based Markers against Bacterial Leaf Blight Disease in Rice Landraces of Yunnan Province of China
Source: Life (Basel). 2023 Oct 23;13(10):2101. doi: 10.3390/life13102101 (PMC10608166; doi:10.3390/life13102101)
Supplement: Supplementary file 1 [file life-13-02101-s001.zip › life-2606900-supplementary.pdf]

Table S1. Distribution of genotypes of 4 bacterial blight resistance genes in rice landraces collected from different regions of Yunnan, China.

Genotypes	Center		Eastern		Northwestern		Southeastern		Southern		Southwestern		Western	
	N	%	N	%	N	%	N	%	N	%	N	%	N	%
GT01	9	18.8	1	33.3	0	0.0	2	9.1	18	15.4	14	12.3	17	17.7
GT02	5	10.4	0	0.0	0	0.0	8	36.4	34	29.1	17	14.9	21	21.9
GT03	1	2.1	0	0.0	0	0.0	0	0.0	3	2.6	0	0.0	0	0.0
GT04	3	6.3	0	0.0	0	0.0	1	4.5	11	9.4	3	2.6	9	9.4
GT05	0	0.0	0	0.0	0	0.0	0	0.0	1	0.9	0	0.0	0	0.0
GT06	0	0.0	0	0.0	0	0.0	0	0.0	0	0.0	1	0.9	2	2.1
GT07	7	14.6	0	0.0	1	25.0	1	4.5	18	15.4	23	20.2	12	12.5
GT08	4	8.3	0	0.0	0	0.0	2	9.1	12	10.3	24	21.1	11	11.5
GT09	1	2.1	0	0.0	0	0.0	0	0.0	1	0.9	0	0.0	0	0.0
GT10	0	0.0	0	0.0	0	0.0	0	0.0	0	0.0	1	0.9	0	0.0
GT11	0	0.0	0	0.0	0	0.0	0	0.0	2	1.7	0	0.0	3	3.1
GT12	0	0.0	0	0.0	0	0.0	0	0.0	0	0.0	1	0.9	1	1.0
GT13	11	22.9	1	33.3	0	0.0	2	9.1	3	2.6	11	9.6	8	8.3
GT14	2	4.2	0	0.0	0	0.0	2	9.1	2	1.7	4	3.5	1	1.0
GT15	4	8.3	0	0.0	0	0.0	0	0.0	1	0.9	2	1.8	2	2.1
GT16	1	2.1	1	33.3	3	75.0	2	9.1	7	6.0	10	8.8	5	5.2
GT17	0	0.0	0	0.0	0	0.0	2	9.1	1	0.9	2	1.8	0	0.0
GT18	0	0.0	0	0.0	0	0.0	0	0.0	0	0.0	1	0.9	0	0.0
GT19	0	0.0	0	0.0	0	0.0	0	0.0	0	0.0	0	0.0	1	1.0
GT20	0	0.0	0	0.0	0	0.0	0	0.0	0	0.0	0	0.0	2	2.1
GT21	0	0.0	0	0.0	0	0.0	0	0.0	1	0.9	0	0.0	0	0.0
GT22	0	0.0	0	0.0	0	0.0	0	0.0	0	0.0	0	0.0	1	1.0
GT23	0	0.0	0	0.0	0	0.0	0	0.0	1	0.9	0	0.0	0	0.0
GT24	0	0.0	0	0.0	0	0.0	0	0.0	1	0.9	0	0.0	0	0.0
Total	48	100.0	3	100.0	4	100.0	22	100.0	117	100.0	114	100.0	96	100.0

Note: N, the number of the landrace with the corresponding *R* gene; %, the percentage of the landrace with *R* gene.
